# About the Distinction between Working Memory and Short-Term Memory

**DOI:** 10.3389/fpsyg.2012.00301

**Published:** 2012-08-23

**Authors:** Bart Aben, Sven Stapert, Arjan Blokland

**Affiliations:** ^1^Department of Neuropsychology and Psychopharmacology, Faculty of Psychology and Neuroscience, Maastricht UniversityMaastricht, Netherlands

**Keywords:** working memory, short-term memory, complex span, simple span, attention, cognitive load

## Abstract

The theoretical concepts short-term memory (STM) and working memory (WM) have been used to refer to the maintenance and the maintenance plus manipulation of information, respectively. Although they are conceptually different, the use of the terms STM and WM in literature is not always strict. STM and WM are different theoretical concepts that are assumed to reflect different cognitive functions. However, correlational studies have not been able to separate both constructs consistently and there is evidence for a large or even complete overlap. The emerging view from neurobiological studies is partly different, although there are conceptual problems troubling the interpretation of findings. In this regard, there is a crucial role for the tasks that are used to measure STM or WM (simple and complex span tasks, respectively) and for the cognitive load reflected by factors like attention and processing speed that may covary between and within these tasks. These conceptual issues are discussed based on several abstract models for the relation between STM and WM.

## Introduction

It is commonly accepted that the storage of new information proceeds through different stages, leading to a permanent storage of information in long-term memory (LTM). Two theoretical models have been proposed with respect to temporary storage: short-term memory (STM) and working memory (WM). STM refers to a cognitive system that is used for holding sensory events, movements, and cognitive information, such as digits, words, names, or other items for a brief period of time (Kolb and Wishaw, 2009). It has been suggested that an average person can hold around seven (Miller, 1956) or four (Cowan, 2001) chunks of information in STM. Although the neural dissociation between STM and LTM, has been questioned (Ranganath and Blumenfeld, 2005) it is clear that the conceptual difference between them lies in the time period for which information is remembered.

The term WM became famous through the homonymic model of Baddeley and Hitch (1974). The WM model has more intrinsic features than the STM model. Based on experimental cognitive psychology research a limited capacity system is assumed that functions as an interface between perception, LTM, and action (Baddeley, 2003). In the classic WM model proposed by Baddeley and Hitch (1974) three different components can be dissociated: a central executive and two domain-specific slave systems used to maintain information (the phonological loop and the visuospatial sketch pad). These memory stores have also been referred to as STM in the multicomponent WM model. The central executive is not a memory system *per se* but instead coordinates the processes of the two slave systems. A new component, the episodic buffer, was later added to the model (Baddeley, 2000).

Despite the different theoretical backgrounds, STM and WM are often used interchangeably and clinical and research literature is blurred by the ambiguous use of both constructs. Many studies acknowledge the coexistence of both STM and WM (e.g., Gathercole and Alloway, 2006; Nadel and Hardt, 2011) but it is also claimed that the term WM has replaced the older term STM (Gray, 2007) or that WM is a theoretical conception of STM (Nairne and Neath, in press). Furthermore, some authors define STM as the ability to maintain information temporarily over periods of seconds (e.g., Neath et al., 2005; Klingberg, 2010), whereas others use this definition to describe WM (e.g., Fletcher and Henson, 2001). According to Baddeley (1992) however, WM is defined as the maintenance and controlled manipulation of a limited amount of information before recall. Most studies use this definition when referring to WM (e.g., Cowan et al., 2005; Ranganath and D’Esposito, 2005; Postle, 2006), yet sometimes no clear distinction between STM (i.e., maintenance) and WM (i.e., maintenance plus manipulation) is made (e.g., Davidson et al., 2006; Jensen et al., 2007). Furthermore, experimental studies on WM often focus solely on the maintenance component of WM (see for example the review by D’Esposito, 2007). Finally, it has also been suggested that both concepts represent the same cognitive process (e.g., Unsworth and Engle, 2007b).

The previous examples illustrate the complex entanglement of terms and definitions that can be encountered in contemporary literature. This problem is further complicated by the lack of consensus on what exactly is WM. Besides the leading model by Baddeley there exist several other models but it is hard to discover commonality between them (Miyake and Shah, 1999). Taking into consideration the enormous amount of literature on STM and/or WM that is being published, this lack of agreement is remarkable. Apparently, the adoption of the terms is ahead of its demarcation. There seems to be a discrepancy between current scientific support for the distinction between STM and WM and the way both terms are used in “every day” science. Whereas some authors may use the terms generically, others clearly refer to two different constructs when discussing STM and WM.

**Figure 1:**
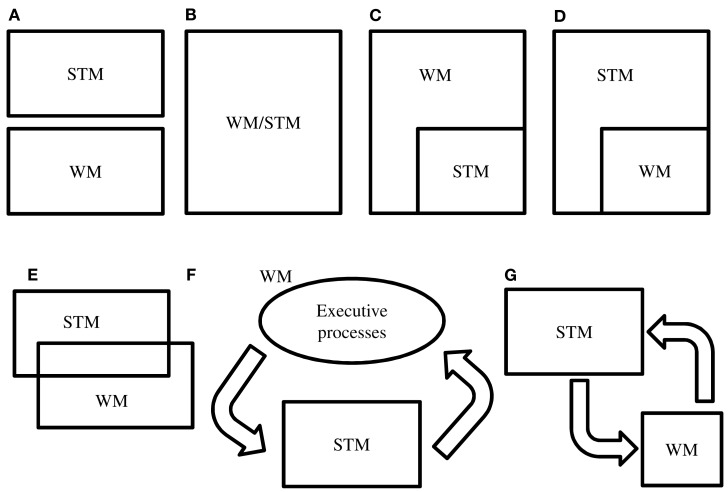
**Hypothetical models of the relation between STM and WM**. There are several ways to hypothesize on the relation between STM and WM. One could consider them as two independent **(A)** or identical **(B)** entities. In models **(C,D)** it is assumed that STM is a part of WM and vice versa. This would imply that there is no transfer of information from WM to STM or from STM to WM. In these models a part of the information in WM is in STM, or a part of the information in STM is in WM. Model **(E)** would not assume a transfer of information from STM to WM (or vice versa) either. Model **(F)** proposes that WM is STM plus additional processes. This model fits the models of Baddeley and Cowan in an abstract way. In model **(G)** it is assumed that information entering STM can be transferred to WM in order to undergo manipulation. After manipulation information is sent back to STM. Model **(G)** considers WM and STM as two different, but strongly collaborative entities. However, the term WM is not appropriate here, since the actual memorizing takes place in the STM component.

In this review, we will discuss the currently largely unnoticed issues on the relationship between STM and WM. The ambiguous use of the constructs is emphasized, which raises the question if they are essentially different. Several models can be proposed in order to illustrate the relation between STM and WM (see Figure 1). Models A, E, and G are conceivable if one assumes STM and WM to be different entities. In case STM and WM cannot be separated then models B, C, D, and F are candidate models. Model F can be considered an abstract display of Baddeley’s WM. Arguments supporting or contradicting the models are discussed throughout the review.

Another important aspect is how we can measure STM and WM. What are the features of an STM or WM task? Clearly, the task-related characteristics of how to measure these concepts relate to their fundamental features. In our opinion these issues have not been dealt with sufficiently yet, and may underlie the confusing use of both constructs. Here we discuss various topics related to STM and WM tasks and the way they may have caused the blurred use of STM and WM.

Overall, this review does not attempt to offer a full overview of the literature on this topic. The goal of this paper is to illustrate conceptual issues with regard to the differentiation between WM and STM and their corresponding tasks with recent evidence from experimental and statistical methodology, and cognitive and neurobiological studies. The article highlights several inconsistencies and aims at increasing the awareness of the ambiguous use of both terms. Before elaborating on these issues, first the basic characteristics of the tasks that are assumed to be measuring STM and WM are discussed.

## Simple Span Versus Complex Span

The finding that a concurrent problem solving task disrupts memorizing a list of items only to a minor extent is an important argument in support of Baddeley’s WM model (Baddeley and Hitch, 1974). Apparently, the two tasks do not interfere much, which is in contradiction with a unitary system for both short-term storage and online processing (i.e., Figure 1B). This finding also underlies the differentiation between the tasks that are used to measure STM and WM (Table 1).

**Table 1:** **Examples of simple and complex span tasks (not exhaustive)**.

Type of memory	Short-term	Working
Type of task	Simple span	Complex span
Nature of task	Tasks which only involve maintenance of items	Tasks which involve maintenance and manipulation of items
Examples	Word span	Reading span
	Digit span	Counting span
	Letter span	Operation span
	Corsi block	Computation span
	Dot memory	*n*-back
	Sentence repetition	Dot matrix
	Knox cube test	Keeping track
	Spatial span	Running memory
	Delayed match to sample	ABCD
		Alphabet
		Mental counters
		Letter rotation
		Loaded word span
		Paced auditory serial addition test
		Mental control
		Serial subtractions
		Mental tracking
		Alpha span
		Letter-number sequencing

*Adapted from Engle et al. (1999), Kane et al. (2004), Lezak et al. (2004), Ackerman et al. (2005), Conway et al. (2005), Colom et al. (2006b)*.

Typical tasks measuring STM are simple span tasks, while WM is commonly measured using complex span tasks. Simple span tasks generally require the participant to maintain a collection of symbols, elements, or spatial positions over a brief period of time (Unsworth and Engle, 2007b). Complex span tasks are created by adding a demanding secondary cognitive task to a simple span task (Unsworth and Engle, 2007b), such as solving mathematical operations (in operation span tasks) or deciding whether a sentence is syntactically or semantically correct (in reading span tasks). Complex span tasks reflect the idea that WM always includes an STM component. This idea is also reflected in models C, E, and F of Figure 1. Just like STM and WM, simple and complex span tasks are often used interchangeably and researchers tend to create new tasks specifically for the purpose of a study. This has led to a proliferation of WM tasks and it is often uncertain to what degree these tasks tap the construct they are supposed to measure. For instance, in a study on capacity limit of STM, a complex span task was created by adding a processing task to a delayed matching to sample task (Todd and Marois, 2004). This way, the authors created a WM task while claiming to be measuring STM. Yet, in other studies intending to measure visual WM, subjects were simply required to retain an image of a face (Courtney et al., 1997) or location of a circle (Klingberg et al., 2002) over a brief period of time. Both these tasks resemble simple span tasks.

The validity of the rather crude distinction between simple span and complex span tasks can also be questioned. There are numerous WM and STM tasks (Table 1), but it is not well known to what degree these diverse tasks tap the same or different constructs (Conway et al., 2005). To state it differently, it is unlikely that these tasks are process pure. STM and WM tasks measure a combination of processes and the extent to which a process affects performance in either domain can differ between tasks. That is, an STM task may reflect the same combination of processes as a WM task, but some of these processes might be addressed more profoundly by STM tasks and others by WM tasks. This is not only a factor between STM and WM task but also within. For instance, some WM tasks clearly include a secondary task competing with information storage. This secondary task can be related (e.g., reading span tasks) or unrelated (e.g., operation span) to the primary task. In other WM tests the secondary task is less clear. The goal of the backward span task for example is to reproduce a sequence of items as in simple span tasks, but this time in the opposite order. Another example is the often used *n*-back task in which the participant is instructed to monitor and continuously update the to-be-remembered information. For example, in the *3*-back task one is required to judge if the displayed element is similar to the element shown three items before. In an attempt to resolve contradictory results, the meta-analysis by Wager and Smith (2003) even disregards this *n*-back task as a manipulation task. In short, this large variability in WM tasks can trouble the differentiation from STM tasks.

Another complicating factor is the overlap with the cognitive domains of information processing speed and attention. Higher order cognitive processes are hierarchically dependent on these basic cognitive processes and different simple and complex span tasks are not always equally demanding when it comes to these functions. The cognitive load of span tasks can vary, for example by increasing the number of to-be-remembered items in STM tasks or by lowering the demands of the secondary task in WM tasks. Complex span tasks involving relatively simple secondary tasks that address only minor processing speed or attention may therefore be more closely related to simple span tasks than complex span tasks involving more demanding secondary operations.

Hence, the distinction between simple and complex span tasks should be conceived with caution. Such a dichotomy suggests a difference between STM and WM, as depicted in Figure 1A. However, the two types of tasks are not process pure; there clearly is a large overlap. In fact, the inclusion of an STM component in WM tasks but not vice versa endorses models C and F in particular, and contradicts models A and D (Figure 1).

## Overlap between Constructs

Since the introduction of the model of Baddeley and Hitch (1974) attempts have been made to explain the characteristics of WM. In Cowan’s recent memory model, WM is considered as an activated portion of LTM. This model includes STM and other central executive processes such as attention that help to make use of STM (Cowan, 2008). On a highly abstract level, this model is comparable to that of Baddeley (Figure 1F). According to Cowan, one reason to pursue the term WM is that performance on WM tasks have been found to correlate with fluid intelligence better than performance on STM tasks. Although the crude distinction between STM and WM tasks may be problematic, there are indications that WM is important for complex cognitive activity, as reflected by correlations with measures of fluid intelligence (e.g., Daneman and Carpenter, 1980; Engle et al., 1999; Conway et al., 2002, 2003; Colom et al., 2005b; Cowan, 2008).

At the same time it has been argued that STM is not as good a predictor of fluid intelligence as WM (e.g., Engle et al., 1999; Conway et al., 2002; Kane et al., 2004). The factors underlying this difference are however unclear. There are attempts made to explain this by inclusion of a third variable. For example, it is shown that WM tasks are often more challenging in terms of control of attention compared to STM tasks (Cowan et al., 2005), and that individual differences in attentional scope are important for individual differences in WM (Kane et al., 2001) and intellectual aptitudes (Cowan et al., 2005). That is, different correlations between STM and WM on the one hand and intelligence on the other hand may not be directly related to substantive differences between STM and WM, but to differences in the attentional demands that the two concepts address. Indeed, both Baddeley (2003) and Cowan et al. (2005) assign an important role for attentional functions in their WM theories and it has been argued that attention is the common factor underlying WM and general intelligence (Conway et al., 2003; Engle and Kane, 2004). This is also included in Figure 1F.

Recently, a change can be observed in cognitive research into the relationship between WM and fluid intelligence. Several studies have argued strongly against the discrepancy between STM and WM for predicting intelligence. For example, Ackerman et al. (2005) reported estimated population correlations between intellectual ability and WM and STM of 0.479 and 0.347 respectively. This is equivalent to 22% shared variance between WM and intellectual abilities and 12% shared variance between STM and intellectual abilities. It is questionable how meaningful such small difference is. In addition, Colom et al. (2006a) conducted a re-analysis of key data sets from studies in favor of the higher predictive power of WM. In this analysis, the STM component that is part of both STM and WM tasks was separated from the additional executive processes tapped only by WM tasks. Hence, all tasks comprised a general STM factor but only the WM task comprised a residual WM factor that was not allowed to correlate with the STM factor. Through this hierarchical approach the authors showed that the STM component of both types of tasks is the critical component contributing to intelligence (Colom et al., 2006a). Furthermore, a large overlap of variance between STM and WM tasks was found after factor analysis of 12 diverse memory span tasks in a sample of 403 participants (Colom et al., 2006b). In fact, 37% of the total amount of variance of all span tasks was explained by one higher order factor and the average loading of the WM and STM measures on this factor was quite similar (0.65 and 0.56, respectively).

These results are supported by Unsworth and Engle (2007b), who conducted a meta-analysis and re-analysis of key data sets. They concluded that simple and complex span have correlations with higher order cognitive functions that are similar in magnitude and that WM and STM tasks are similarly affected by several experimental variables such as the phonological similarity between list items. Furthermore, they stated that both constructs are similar in terms of performance indicators. Unsworth and Engle suggested that the variance common to simple and complex span tasks is responsible for their predictive power and reject the notion that STM and WM are largely different constructs. Thus, according to this study, simple and complex span tasks are likely to measure similar processes (i.e., are not process pure) but differ in the extent to which these processes operate in a particular task (Unsworth and Engle, 2007b).

Another study also demonstrated that WM and STM are hardly distinguishable and even proposed that fluid intelligence is nearly perfectly correlated to both constructs (Martinez et al., 2011). Consistent results were obtained in children aged 5–7 years. Equally strong correlations between STM and fluid intelligence (*r* = 0.52) and WM and fluid intelligence (*r* = 0.59) were found, suggesting a shared variance of STM and WM that can predict intelligence (Hornung et al., 2011). Finally, STM has also been identified as the construct accounting for the relationship between complex span measures and reasoning (Krumm et al., 2009).

In general, these studies show that by evaluating the contribution of STM performance to intelligence prior to the contribution of WM performance, the residual variance in WM performance is not or only weakly associated with intelligence (e.g., Colom et al., 2005a,b, 2008). Since models of WM usually include STM (for example Baddeley, 2000; Cowan, 2008) this may not be surprising. Yet these findings again raise the question to what degree both constructs differentiate and whether both WM and STM measures are not better explained by one general factor. If WM performance cannot explain variance in intelligence in addition to STM performance, one could consider STM and WM as similar concepts, as Unsworth and Engle (2007b) also claim. In that case, model B is correct (Figure 1).

There are several theories that try to identify the factors responsible for the correlation between performance on WM tasks and cognitive abilities. Colom et al. (2006a) proposed that the higher processing requirements of complex span tasks are crucial. The concurrent processing required to solve the additional task may use some of the capacity otherwise used for the storage of information. This results in a diminished reliability of the stored information and a decrease of performance. In other words, persons with more general cognitive capacity, as reflected by fluid intelligence, will perform better on WM tasks. This is quite different from the idea that complex span tasks measure something additional to simple span tasks, such as attention or information processing speed. However, it is not clear to what process the term cognitive load is referring. Barrouillet et al. (2007) argue that the duration of the intervening tasks is the most important factor. According to their resource sharing model the cognitive load of a complex span task is determined by the proportion of time that is spend on the secondary task. That is, attention is captured from the original task during the performance of the interfering activity, resulting in a decay of the to-be-remembered items. The longer the attention is switched away from the memory task and captured by concurrent activities, the stronger the decrease of WM performance (Barrouillet et al., 2007). This would also imply that the cognitive load of STM tasks is depending on internal distractors, since there are no external distractors on these tasks.

Unsworth and Engle (2007a) reintroduced the terms primary and secondary memory to offer another explanation for individual differences in WM performance. First, they argue that an immediate free recall task can be used to measure WM capacity and is an equally good predictor of higher order cognitive functions as are complex span tasks. Next, they suggest that performance on this task relies on two factors: maintaining performance in primary memory and effectively searching for representations that have been displaced from primary to secondary memory (Unsworth and Engle, 2007a). Primary memory is considered a limited capacity short-term component. The maintaining process in this store depends on general processes that are also described in other models, such as focus of attention (Cowan, 2008) or general storage capabilities (Colom et al., 2006a). Information is displaced from primary to secondary memory when primary memory is fully occupied or when attention is disengaged from the maintained items, for example when performing a distracting secondary task. According to this model, individuals that perform low on WM tasks are either impaired at maintaining information in primary memory (i.e., the short-term component) or are more likely to have their attention captured by distraction (Unsworth and Engle, 2007a; Unsworth et al., 2010). As demonstrated by Unsworth et al. (2010), this dual-component model is both applicable on the classic STM tasks (e.g., immediate free recall) and WM tasks. However, the effects may be more profound on WM tasks, because of the larger distraction.

In the end, these explanations are comparable to a large extent. The difference between the views is that some consider cognitive load to be a general exhaustible capacity (Colom et al., 2006a), while others emphasize the importance of attentional processes (Barrouillet et al., 2007; Unsworth and Engle, 2007b; Cowan, 2008). Clearly, the original claim that WM is a better predictor of intelligence than STM is under attack. There is substantial evidence for a large association between intelligence and both WM and STM performance, and the underlying construct accounting for this association may be simple short-term storage, attentional processes, or both. These findings make it hard to separate STM and WM and their corresponding tasks. A better approach may be to consider the different tasks as part of a continuum with variations in the mentioned factors. This would imply that model B (Figure 1) is a valid description of the relation between STM and WM.

## Neurobiological Findings

According to most models, STM is a critical component of WM (e.g., Baddeley, 2000; Cowan, 2008), a notion that is also reflected in the design of STM and WM tasks. One of the brain regions primarily related to WM and STM is the prefrontal cortex (PFC), in particular the dorsolateral part (dlPFC). For instance, patients with isolated lesions in the dlPFC typically show impairment on delayed-response tasks that require an active maintenance of information (Gazzaniga et al., 2009) and imaging studies have related the delay-period of memory tasks to activity in the PFC (e.g., Narayanan et al., 2005; Zarahn et al., 2005). In addition to the dlPFC there are several other regions linked to WM. Imaging studies have revealed activity during execution of the *n*-back task in the dlPFC and ventrolateral PFC (vlPFC), lateral premotor cortex, dorsal cingulate and medial premotor cortex, frontal poles, and medial and lateral posterior parietal cortex (PPC; Owen et al., 2005). These regions constitute a complex distributed cortical network involved in activation and allocation of resources (Khan and Muly, 2011). Because the components of maintenance and particularly manipulation are hard to isolate in the *n*-back task, it is impossible to identify which regions in this extensive network correspond to either WM or STM. Yet, such dissociation is desirable to support a distinction between both processes. To demonstrate a difference in neuronal activation related to both concepts it is necessary to design tasks that can isolate activity related to the manipulation component of WM from activity linked to maintenance. Because it is beyond the scope of this article to discuss all brain structures linked to STM and WM the remaining of this section will focus mainly on the dlPFC and its relation to maintenance and manipulation.

First, it is important to understand that issues similar to the ones discussed in the previous two paragraphs can also be encountered in imaging studies. For example, it has been shown that activity in the dlPFC is higher during the performance of a manipulation task (i.e., reordering a sequence of letters into alphabetical order) relative to performance of a maintenance task (i.e., retaining a sequence of letters; D’Esposito et al., 1999). This seems to be neurobiological support for a distinction between WM and STM. If we indeed consider STM and WM as two separate systems than the question arises why dlPFC activation was also observed during the maintenance of information. Since the dlPFC is also critically involved in attention (Kane et al., 2001), a more parsimonious explanation would be that the increase in activity of this region simply reflected an increase in cognitive load because the manipulation task was more demanding than the maintenance task (D’Esposito et al., 1999). Indeed, there are studies showing that increased activity in dlPFC corresponds to an increased maintenance load (Veltman et al., 2003; Narayanan et al., 2005). For example, it was shown that variation in maintenance and manipulation load were both related to dlPFC activity and that both tasks tap virtual identical systems (Veltman et al., 2003). These findings are also in agreement with models that claim that attentional capacities are critical for both WM and intelligence (Conway et al., 2003; Engle and Kane, 2004). That is, WM tasks in general consist of two interleaved tasks and thus require more dividing of attention reflected by dlPFC activity compared to STM tasks. Others have suggested that the relationship between WM and fluid intelligence can be partly explained by interference control. Burgess et al. (2011) showed that activation during the *n*-back task correlated with activation during a set of WM span and fluid intelligence tasks. These activation patterns were centered on the dlPFC and parietal cortex and reflected the common dependence on interference control during performance of the tasks. However, as acknowledged by the authors, it was unclear to what degree interference control independently related to processing or storage because the WM tasks used in this study are measuring both (Burgess et al., 2011).

Another interesting line of research used transcranial magnetic stimulation (TMS) to define the PFC-related processes linked to the delay-periods of WM and STM tasks. Studies have shown that administration of repetitive TMS (rTMS) on the PFC did not impair performance on STM tasks requiring maintenance of verbal (Feredoes et al., 2007) or spatial (Hamidi et al., 2008) information. Postle et al. (2006) used rTMS to disentangle brain activity crucially involved in maintenance and manipulation. First, they instructed participants to either maintain or alphabetize a sequence of letters. On the maintaining trials subjects had to reproduce a sequence of letters in the same order, whereas on the alphabetize trials they had to reorder the letters in alphabetical order (i.e., maintain plus manipulate the information). Analysis of the fMRI activity revealed that manipulation-related activity was independent of maintenance-related activity in both the dlPFC and superior parietal lobule (SPL). In the second part of the study, subjects performed the same tasks again, but this time rTMS pulses were administered to the dlPFC and SPL. This procedure yielded a different result. rTMS on the dlPFC selectively disrupted manipulation but not maintenance. In other words, these results are consistent with a model of segregation of manipulation from maintenance functions in the PFC (Postle et al., 2006). In agreement with that, Postle argues that the PFC activity observed during WM tasks is not related to short-term retention but to control processes not exclusively limited to WM (Postle, 2006). According to this view, WM is not a specialized system but an emerging property arising through the coordinated recruitment of different brain systems. The PFC is involved in controlling this process but not in storing information. From this perspective, the control of WM is not qualitatively different from the control of any other behavioral or mental function which is in agreement with the large diversity of functions linked to PFC activation (Postle, 2006). This is also in accordance with a study showing an rTMS interference effect on the dlPFC for the *2*-back task but not for the *1*-back task (Sandrini et al., 2008). In this study it was assumed that the *1-*back task measures maintenance and the *2*-back task measures maintenance plus manipulation. One can question the validity of this assumption but nevertheless these results also hint at a controlling function of the dlPFC instead of a storing function. Finally, findings from an imaging study also suggested that increased activity in the medial part of the dlPFC during WM tasks is related to the monitoring of information that is being manipulated (Champod and Petrides, 2007).

The commonality between WM and intelligence has also been studied from a neurobiological perspective. Colom et al. (2007) demonstrated an overlap of gray matter intensities correlating to measures of general intelligence and WM capacity. They only found small overlap in the dlPFC but identified the right superior frontal gyrus and left middle frontal gyrus and, to a lesser degree, the right inferior parietal lobule as the common anatomic framework for WM and general intelligence. Remarkably, in this study the forward and backward digit span test were used to measure WM. This choice is surprising because, as acknowledged by the authors, it is far from clear to which extent these tasks measure WM or STM. The forward digit span in particular has been appointed to the class of simple span tasks (Engle et al., 1999; Colom et al., 2005b) and there is no consensus about the processes involved in the backward span (Richardson, 2007). The latter requires the transposition of order which may be considered as the additional task in a complex span task (Hornung et al., 2011) but it has also been claimed that the backward span task belongs to the class of simple span tasks (e.g., Rosen and Engle, 1997; Engle et al., 1999). Colom et al. (2007) argue that the forward and backward test overlap from a behavioral as well as biological perspective. In other words, the tasks do not specifically tap either STM or WM. Following this reasoning, the neuroanatomical overlap between WM and intelligence that is demonstrated in this study is identical to that between STM and intelligence.

Finally, there are also claims that increased dlPFC and vlPFC activity is elicited by encoding strategies (e.g., chunking) and that increasing the cognitive load of a WM task can result in a change of strategy, which may reflect the increase in dlPFC activation (Bor et al., 2004). In line with this, another study demonstrated differential involvement of the left and right PPC for *1* and *2*-back tasks. This finding might also have to do with a switch of strategy (Sandrini et al., 2012).

In sum, whereas the dlPFC used to be broadly associated with the delay-period of span task, there is now evidence for a more specific role of it in manipulating information or controlling other brain structures. At the same time, the role of dlPFC in the maintenance of information has been disputed. There are studies that suggest that dlPFC activity is linked to the processes that are required on WM tasks in addition to short-term storage, such as attention, strategy use, or general cognitive capacity. This is in accordance with models that claim that WM is composed of STM plus additional processes. Models C and in particular F (Figure 1) meet this criterion, although according to Postle’s view far more functions and brain regions are involved. It is however not in agreement with theories stating that simple and complex span tasks basically measure the same processes, as displayed in model B of Figure 1 (e.g., Ackerman et al., 2005; Colom et al., 2006b; Unsworth and Engle, 2007a). These theories are based on the overlap in variance between STM and WM on the one hand and intelligence on the other hand. Model B also seems to be supported by structural imaging findings. One important factor that complicates the interpretation of these findings is the fact that many different span tasks are used to measure STM or WM. There is still debate on the validity of these tasks and differences between studies may be explained by differences between the used tasks. An additional complicating factor is that maintenance (supposedly STM) cannot easily be dissociated from manipulation (supposedly WM) because manipulation also entails the maintenance of information. Taken together, this suggests that more consensus is needed in using memory tasks to further understand the localization of maintenance and manipulation processes in the brain.

## Conclusion

The interchangeable use of STM, WM, simple span tasks, and complex span tasks encountered in contemporary literature indicates that the differentiation between STM and WM is far from clear. Although they may be conceptually distinctive, the interchangeable use of STM and WM is more or less understandable since there clearly is a large overlap between both. So far, studies using correlational designs have not consistently succeeded to unequivocally differentiate between STM and WM. There are in fact strong arguments for a large or even complete overlap of both constructs, which is in favor of model E or even B (Figure 1). Model B could be a valid model if one assumes that only other factors (e.g., cognitive load, attention, processing speed) mediate the difference between simple and complex span tasks. However, this model does not take into account that STM and WM are theoretically different cognitive entities. In this regard, models C and E are a more accurate display. In models C and D it is assumed that there is no transfer of information from WM to STM or from STM to WM. Model C is more likely than model D. It is hard to imagine a model in which WM is a part of STM, while there are claims that WM consists of STM plus additional processes. Model F also fits with this idea. It is also supported by recent neurobiological findings. Executive processes such as attention (i.e., the central executive) direct information into a short-term storage slave in order to maintain the information. The dlPFC is a candidate for the neural correlate of these processes and rTMS studies concerning this region favor a dissociation between maintenance and manipulation. Model (G) considers WM and STM as two different, but strongly collaborative entities. However, the term WM is not appropriate here, since the actual memorizing takes place in the STM component. Finally, model A is not a valid model. From the fact that complex span tasks and WM models always include an STM component it follows that STM and WM are no independent processes.

An important issue to consider here is the fact that definitions of STM and WM depend on the tasks that are used to measure both constructs. At present, the differentiation of simple and complex span tasks is in accordance with WM models that include STM components (i.e., models C and F of Figure 1). The large variety of simple and complex span tasks (which also vary in cognitive load) however troubles the distinction between STM and WM. There are no standard STM or WM tasks, which makes it hard to compare different studies and tasks. Furthermore, the intimate relation between STM and WM justifies the question whether the two concepts are largely identical or whether the derived STM and WM tasks are simply incapable of differentiating between them. Ignoring this question by equating WM with STM would be an easy way out but would not reflect the current use of this terminology. The two terms are used in innumerable scientific and clinical reports and different definitions and tasks are often assigned to both constructs. Although in some papers the two terms make no differences for the account offered, in others an actual difference is made, indicating that there are reasons to suppose substantive differences between both. As Unsworth and Engle (2007b) rightly point out, the distinction between STM and WM is not only important from a purely cognitive perspective but also because of the psychological batteries that rely on the tasks that are assumed to measure these constructs. Furthermore, memory research in several areas is depending on such tasks and many neurocognitive and neurobiological studies aim at relating brain structures and biological processes to either STM or WM. A non-uniform use of terminology and tasks may lead to inconsistencies between interpretations and conclusions of such studies. Hence, it is highly important to be aware of the scientific debate on the demarcation of STM and WM and to eventually reach consensus on this issue.

Independent of the memory model one supports, it is important to realize that there may be several variables that mediate the difference between STM and WM. As opposed to simple span tasks, complex span tasks often address a higher cognitive load and differences in task performance may be explained by differences in short-term storage capacity, attentional demands, processing speed, or strategy. However, it can be questioned whether the variations in these factors justify the substantive distinction between STM and WM. There probably not only exists a difference in demands between simple and complex span tasks but also within. A difficult task can in some cases require a different kind of processing than a simple one. For example, subjects may choose to apply strategies such as chunking, visualizing, or categorizing items in hard tasks, while they do not use these strategies when asked to simply recall a single sequence of digits. Hence, it is not *per se* the task that is decisive for the type of memory used but the process applied by the subject. In case a subject adds meaning to the to-be-remembered items or exercises some other manipulation to the information, the task would be measuring more WM than STM. In other words, a parsimonious explanation for the differences found between simple and complex span performance might be that some tasks (e.g., complex span) are just more demanding than others (e.g., simple span) and therefore require different or additional processing.

To solve this issue, there is a need for more studies that vary the cognitive load within maintenance and manipulation tasks. That way, differences in task performance can be validly ascribed to task differences or load differences, or both. Whether the load reflects attention, STM, or other (combinations of) cognitive processes remains open for debate. Meanwhile, a pragmatic solution to the illustrated problem would be to specify both STM and WM tasks in terms of duration (e.g., seconds) and processing load (e.g., number of items or characteristics of the task). This way, tasks can be easier compared in terms of cognitive complexity. Furthermore, to clarify the jumble of STM and WM tasks, consensus should be reached on what constitutes typical STM tasks and what typical WM tasks. These conditions are important to untangle the complexity of STM and WM concepts.

## Conflict of Interest Statement

The authors declare that the research was conducted in the absence of any commercial or financial relationships that could be construed as a potential conflict of interest.
